# Development and Validation of Two Self-Reported Tools for Insulin Resistance and Hypertension Risk Assessment in A European Cohort: The Feel4Diabetes-Study

**DOI:** 10.3390/nu12040960

**Published:** 2020-03-30

**Authors:** Spyridon Kanellakis, Christina Mavrogianni, Kalliopi Karatzi, Jaana Lindstrom, Greet Cardon, Violeta Iotova, Katja Wikström, Samyah Shadid, Luis A. Moreno, Kaloyan Tsochev, Éva Bíró, Rumyana Dimova, Emese Antal, Stavros Liatis, Konstantinos Makrilakis, Yannis Manios, on behalf of the Feel4Diabetes-study group

**Affiliations:** 1 Department of Nutrition and Dietetics, School of Health Science and Education, Harokopio University, 17671 Athens, Greece; kanellakis@hua.gr (S.K.); cmavrog@hua.gr (C.M.); pkaratzi@hua.gr (K.K.); 2 Department of Public Health Solutions, Finnish Institute for Health and Welfare, 00271 Helsinki, Finland; jaana.lindstrom@thl.fi (J.L.); katja.wikstrom@thl.fi (K.W.); 3 Department of Movement and Sports Sciences, Faculty of medicine and Health Sciences, Ghent University, 9000 Gent, Belgium; Greet.Cardon@ugent.be; 4 Department of Paediatrics, Medical University Varna, 9002 Varna, Bulgaria; violeta.iotova@mu-varna.bg (V.I.); kalooyan@abv.bg (K.T.); 5 Department of Endocrinology, Ghent University Hospital, 9000 Gent, Belgium; samyah.shadid@uzgent.be; 6 Growth, Exercise, Nutrition and Development Research Group, School of Health Sciences, University of Zaragoza, 50009 Zaragoza, Spain; lmoreno@unizar.es; 7 Instituto Agroalimentario de Aragón (IA2), 50009 Zaragoza, Spain; 8 Instituto de Investigación Sanitaria de Aragón (IIS Aragón), 50009 Zaragoza, Spain; 9 Centro de Investigación Biomédica en Red de Fisiopatología de la Obesidad y Nutrición (CIBERObn), Instituto de Salud Carlos III, 28029 Madrid, Spain; 10 Division of Health Promotion, Department of Preventive Medicine, Faculty of Public Health, University of Debrecen, 4032 Debrecen, Hungary; biro.eva@sph.unideb.hu; 11 Department of Diabetology, Clinical Center of Endocrinology, Medical University Sofia, 1431 Sofia, Bulgaria; dr.roumyana.dimova@gmail.com; 12 Hungarian Society of Nutrition, 1088 Budapest, Hungary; diet.Emese.Antal@gmail.com; 13 National and Kapodistrian University of Athens Medical School, 11527 Athens, Greece; s.liatis@yahoo.com (S.L.); kmakrila@med.uoa.gr (K.M.)

**Keywords:** European IR Risk Index, European HTN Risk Index, screening, type 2 diabetes, hypertension

## Abstract

Early identification of type 2 diabetes mellitus (T2DM) and hypertension (HTN) risk may improve prevention and promote public health. Implementation of self-reported scores for risk assessment provides an alternative cost-effective tool. The study aimed to develop and validate two easy-to-apply screening tools identifying high-risk individuals for insulin resistance (IR) and HTN in a European cohort. Sociodemographic, lifestyle, anthropometric and clinical data obtained from 1581 and 1350 adults (baseline data from the Feel4Diabetes-study) were used for the European IR and the European HTN risk assessment index respectively. Body mass index, waist circumference, sex, age, breakfast consumption, alcohol, legumes and sugary drinks intake, physical activity and sedentary behavior were significantly correlated with Homeostatic Model Assessment of IR (HOMA-IR) and/or HTN and incorporated in the two models. For the IR index, the Area Under the Curve (AUC), sensitivity and specificity for identifying individuals above the 75th and 95th of HOMA-IR percentiles were 0.768 (95%CI: 0.721–0.815), 0.720 and 0.691 and 0.828 (95%CI: 0.766–0.890), 0.696 and 0.778 respectively. For the HTN index, the AUC, sensitivity and specificity were 0.778 (95%CI: 0.680–0.876), 0.667 and 0.797. The developed risk assessment tools are easy-to-apply, valid, and low-cost, identifying European adults at high risk for developing T2DM or having HTN.

## 1. Introduction

Despite the vast scientific research and the numerous health initiatives indicating that Type 2 Diabetes Mellitus (T2DM) is preventable, its prevalence is constantly rising. At the moment, 483 million people suffer from diabetes around the world. Although Europe holds the second lowest prevalence of 6.3%, it is expected to increase to 7.3% and 7.8% by 2030 and 2045, respectively [1]. Similarly, hypertension (HTN), which is the strongest risk factor for all-cause mortality globally [2], keeps on rising and affects one in four adults [3].

The importance of early identification of both T2DM and HTN has been well-documented [4,5,6,7]. The diagnosis of T2DM includes the assessment of fasting plasma glucose (FPG), 2-h plasma glucose during an oral glucose tolerance test, or glycated hemoglobin (HbA1c) measurement [8]. Furthermore, according to the European Society of HTN, measurement of blood pressure in various conditions, such as at the office or at home, or daytime versus night-time, determines its classification from optimal blood pressure (BP) to grade 3 HTN [9]. However, since people with HTN have no symptoms, they do not monitor their BP. Consequently, as shown in a recent review, 5–10% of subjects with stage 2 HTN are undiagnosed or untreated [10]. Either because of the cost of blood tests, the practical arrangements of being measured or the lack of symptoms, both for insulin resistance (IR) and high BP, many people do not perform regular checks and, consequently, diagnosis is only made often after severe complications have developed over time [11]. Therefore, the application of low-cost and easy-to-apply methods for the early diagnosis of pre-diabetes and HTN could be a useful tool for public health [12].

There is a growing number of screening tools assessing glycaemic and BP status via the incorporation of various risk factors such as age, history of disease, sex, ethnicity, body mass index (BMI), medication, etc. [13,14,15,16,17]. Regarding the manifestation of T2DΜ, there are many risk scores and indices in the literature. However, most of them incorporate measures of blood glucose, which is not always feasible to be measured, especially in populations of low socioeconomic status. Additionally, none of them has been developed based on a multiethnic population. Moreover, most of them do not take into account dietary habits as predictors [13,15,18,19]. Nonetheless, there is no risk score identifying IR. Impaired insulin sensitivity represents early abnormalities in glycaemic control and constitutes a great predictive index for T2DM [20,21]. In addition, the impact of IR on the capacity metabolic roles of liver, adipose tissue and skeletal muscle is detrimental for human health [22]. As for HTN, there are some prediction models developed, but their application does not suit to all ethnicities and races [23], age groups [14], or incorporate, as well, BP measurements [16]. This is impractical for many people as they do not regularly measure their BP, or it is misleading, as the reported BP measurement does not represent an accurate estimation due to lack of proper assessment according to the guidelines (i.e., standard conditions and measurements during three different visits) [24].

The aim of the current study was to develop and validate two risk assessment indices for the identification of adults with IR (European IR Risk Index) and grade 2 and 3 HTN (European HTN Risk index) via demographic, anthropometric, dietary and lifestyle parameters in a large European cohort.

## 2. Materials and Methods

### 2.1. Study Background

The current study was based on the baseline data retrieved from the EU-funded Feel4Diabetes-study, which intended to design, apply and evaluate an intervention program in schools and communities in order to prevent T2DM among families across Europe. The Feel4Diabetes-study was registered at clinicaltrials.gov as NCT02393872.

### 2.2. Ethics Approval and Consent to Participate

The Feel4Diabetes-study adhered to the Declaration of Helsinki and the conventions of the Council of Europe on human rights and biomedicine [25]. All participating countries obtained ethical clearance from the relevant ethical committees and local authorities. More specifically, in Belgium the study was approved by the Medical Ethics Committee of the Ghent University Hospital (ethical approval code: B670201524437); in Bulgaria, by the Ethics Committee of the Medical University of Varna (ethical approval code: 52/10-3-2016r) and the Municipalities of Sofia and Varna, as well as the Ministry of Education and Science local representatives; in Finland, by the hospital district of Southwest Finland ethical committee (ethical approval code: 174/1801/2015); in Greece, by the Bioethics Committee of Harokopio University (ethical approval code: 46/3-4-2015) and the Greek Ministry of Education; in Hungary, by the National Committee for Scientific Research in Medicine (ethical approval code: 20095/2016/EKU); and in Spain, by the Clinical Research Ethics Committee and the Department of Consumers’ Health of the Government of Aragón (ethical approval code: CP03/2016). All participants gave their written informed consent prior to their enrolment in the study.

### 2.3. Study Protocol and Recruitment

A detailed description of the methodology of the Feel4Diabetes-study has been previously published [26]. Briefly, the recruitment of the population study was carried out via a multi-stage sampling procedure in selected provinces of six European countries (Hungary, Bulgaria, Finland, Belgium, Greece and Spain). In each country, primary schools located in the selected municipalities were used as the entry-point to the community and parents having children attending the first three grades were invited to complete the Finnish Diabetes Risk Score (FINDRISC) and a brief questionnaire on lifestyle habits (self-reported data). If at least one parent fulfilled the country- specific cut-off point for FINDRISC (for the majority of countries that was set as a FINDRISC score ≥ 9), both parents (regardless their individual FINDRISC score) were invited to undergo a brief medical check-up. This procedure led to a cohort with a wide distribution of FINDRISC values among the participating parents, i.e., 25.9%, 37.3% and 36.8% of participants had a total FINDRISC score <9, 9–11 and ≥12 respectively.

Exclusion criteria for the development of the two risk assessment indices were: previous diabetes diagnosis, not following the fasting protocol, current antihypertensive treatment and incomplete data. Therefore, from the initial 3153 parents, 1572 were excluded from the Homeostatic Model Assessment of IR (HOMA-IR) index and 1786 were excluded from the HTN index, and this resulted in 1581 (Bulgaria, *n* = 367; Finland, *n* = 218; Belgium, *n* = 214; Greece, *n* = 476; and Spain, *n* = 306) and 1350 (Hungary, *n* = 19; Bulgaria, *n* = 361; Finland, *n* = 278; Belgium, *n* = 173; and Greece, *n* = 519) subjects for each model, respectively. Insulin data were not analyzed in Hungary, thus the data obtained from this country was not included the IR risk index. Similarly, the data obtained from Spain was excluded from the HTN risk index, as alcohol intake was not recorded in this cohort. Blood pressure and blood indices (i.e., insulin and glucose) were measured by trained researchers using standardized procedures and calibrated equipment in every country as described elsewhere [26]. All variables used in the two developed tools were self-reported (including anthropometric measurements).

### 2.4. Measures

Questionnaire data: All the relevant data, such as sociodemographics (i.e., sex, age, educational level, marital status, etc.), behavioral indices regarding dietary habits, physical activity and sedentary behaviors (i.e., portions of sugary drinks per week, number of meals and snacks during a day, minutes of daily vigorous physical activity, time spent in front of computers and television, etc.) were collected from participants [26].

Anthropometry: All study participants received paper measuring tapes and brief written instructions on how to measure their height, weight and waist circumference. BMI and waist circumference were classified based on the World Health Organization (WHO) criteria [27].

BP measurement: BP was measured on the right arm, in a sitting position using electronic sphygmomanometers (OMRON M6 or OMRON M6 AC, Omron Healthcare, Kyoto, Japan) after five minutes of rest, on three occasions, at one-minute intervals. Participants were classified according to the European guidelines [28] in the following categories: optimal (systolic BP < 120 mmHg and/or diastolic BP < 80 mmHg), normal (systolic BP 120–129 mmHg and/ or diastolic BP 80–84 mmHg), high normal (systolic BP 130–139 mmHg and/or diastolic BP 85–89 mmHg), grade 1 HTN(systolic BP 140–159 mmHg and/ or diastolic BP 90–99 mmHg), grade 2 HTN (systolic BP 160–179 mmHg and/or diastolic BP 100–109 mmHg), and grade 3 HTN (systolic BP ≥ 180 mmHg and/or diastolic BP ≥ 110 mmHg), with the BP category to be defined by the highest level of BP, either systolic or diastolic.

Blood indices: Blood samples were drawn in the morning after overnight fasting (duration: eight hours or longer). FPG and fasting insulin was analyzed in accredited laboratories, using similar enzymatic assays in all study centers. HOMA-IR was calculated as indicated by Matthews et al. [29].

### 2.5. Statistical Analysis

Factors associated with HOMA-IR and HTN were identified by Pearson’s or Spearman’s correlation and analyses of variance. For dietary behavior and physical activity factors, receiver operating characteristic (ROC) analysis was performed and the optimal cut-offs were determined by the point with the shortest distance to (0,1) in the ROC curve that maximizes the sensitivity (Se) and specificity (Sp) of the test. The distance for each observed cut-off was calculated as the square root of [(1 − Se)^2^ + (1 − Sp)^2^] [30]. For other variables, such as waist circumference and BMI the cut-off values set by WHO were used. The population was randomly separated to 2/3 and 1/3 for the development (*n* = 1076 IR cohort and *n* = 906 HTN cohort) and validation (*n* = 505 IR cohort and *n* = 444 HTN cohort) of the indices respectively. For the development of the risk assessment indices, backward linear regressions were performed, with dependent variables the percentiles of HOMA-IR and 5 categories of HTN classification, respectively. The exclusion criteria of independent variables were set at *p* > 0.10. The adjusted β-coefficients were multiplied and rounded to the nearest integer value as needed so as to sum up to 40 points. In order to validate the indices, ROC analysis was performed to the validation cohort. The scores with the best combination of Se and Sp (determined as described previously), for 75th and 95th percentile for HOMA-IR, and for grade 2 and 3 HTN, respectively, were used as cut-off scores for the interpretation of the results. The statistical analyses were performed using the Statistical Package for Social Sciences (SPSS Inc., Chicago, IL, USA), version 21.0.

## 3. Results

The descriptive characteristics of the study population for the European IR Risk Index and the European HTN Risk Index are presented in Table 1. Moreover, the sex distribution was 33.8% males and 66.2% females and 31.9% males and 68.1% females, for European IR Risk Index and the European HTN Risk Index, respectively. None of the aforementioned variables differed significantly between development and validation cohorts.

Regarding the European IR Risk index, the parameters found to be significantly associated with the HOMA-IR were: number of breakfast occasions per week, unsweetened and sweetened milk consumption, sugary drinks consumption, fish, red meat, fruits and vegetables consumption, BMI, sex, waist circumference, number of walking sessions during the week lasting at least 30 min, number of vigorous physical activity sessions during the week lasting at least 10 min and leisure screen time (i.e., television, video games, computers, etc.). At the final model the variables found to be statistically significant in identifying the HOMA-IR percentiles after the stepwise procedure were BMI, screen time, sex, breakfast, sugary drinks, waist circumference, walking and vigorous physical activity as shown in Table 2.

As for the European HTN Risk Index the parameters found to be significantly associated with the BP categories as determined by the ESH were: number of cigarettes per day, number of lunch occasions per week, morning snacks per week, afternoon snacks per week, evening snacks per week, whole grain foods consumption (i.e., whole wheat bread, breakfast cereals), fruits and vegetables, red meat, white meat, fish, nuts and legumes consumption, alcohol intake, and leisure screen time (i.e., television, video games, computers, etc.). At the final model the variables that were found to be statistically significant in identifying grade 2 and 3 HTN after the stepwise procedure were legumes consumption, alcohol intake, sex, age, BMI, and vigorous physical activity, as shown in Table 3.

**Figure 1:**
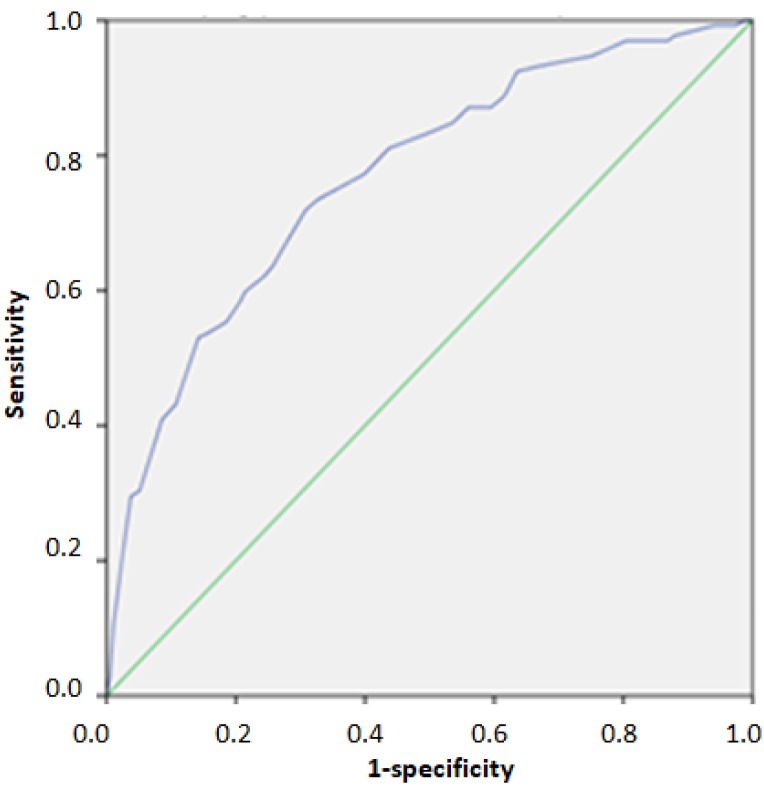
ROC Curve for identifying individuals above the 75th percentile of HOMA-IR.

**Figure 2:**
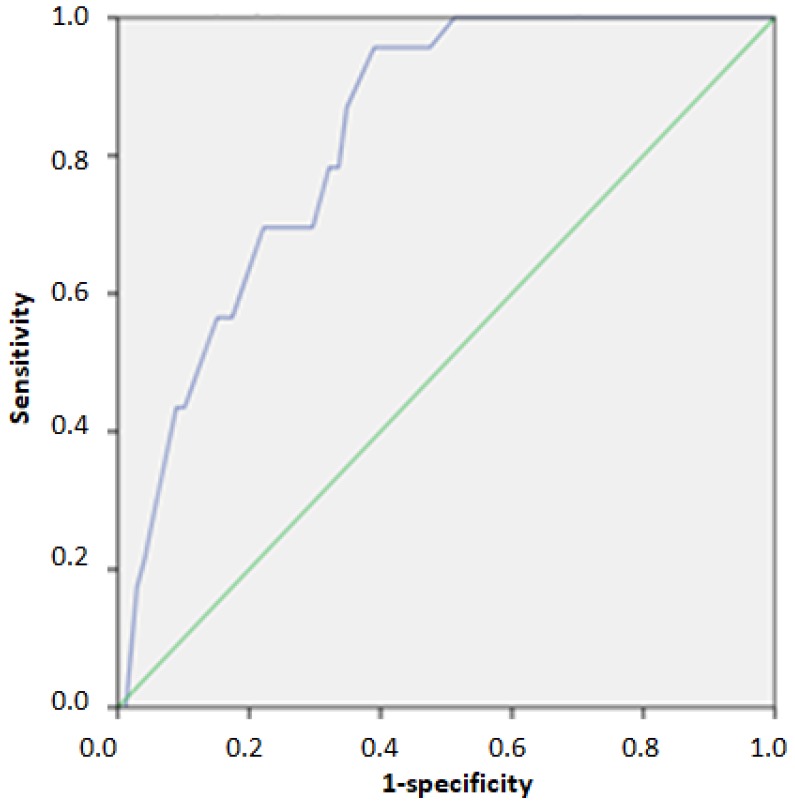
ROC Curve for identifying individuals above the 95th percentile of HOMA-IR.

**Figure 3:**
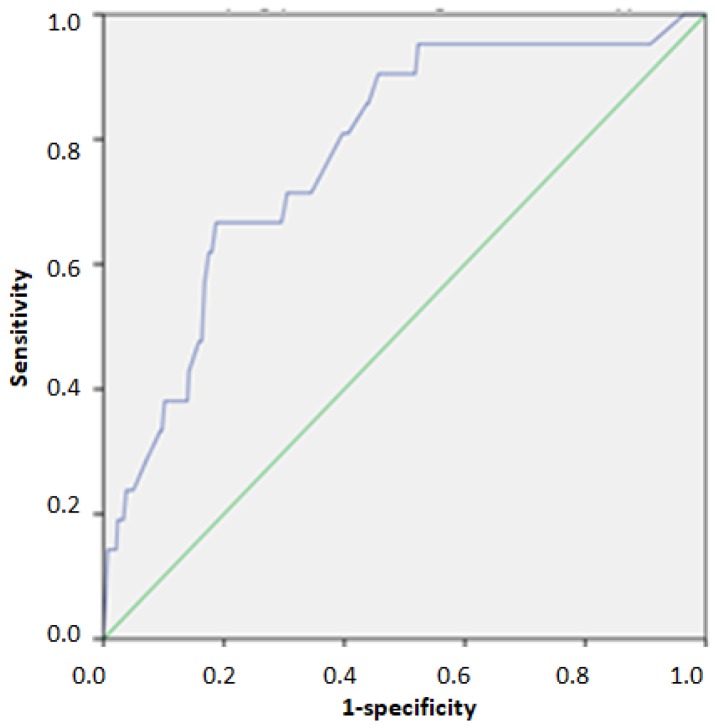
ROC Curve for identifying individuals with grade 2 and 3 hypertension.

The ROC analysis in the validation cohort indicated an area under the curve (AUC) 0.768 (95%CI: 0.721–0.815) for identifying individuals above the 75th percentile of HOMA-IR. The index cut-off score for identifying individuals above the 75th percentile was 23/40, as indicated by the optimal match of Se and Sp (0.720 and 0.691, respectively) (Table 4, Figure 1). Furthermore, the indicated AUC for identifying individuals above the 95th percentile of HOMA-IR was 0.828 (95%CI: 0.766–0.890). The index cut-off score for identifying individuals above the 95th percentile was 31/40, as indicated by the optimal match of Se and Sp (0.696 and 0.778, respectively) (Table 4, Figure 2). Regarding HTN, the ROC analysis in the validation cohort indicated an AUC 0.778 (95%CI: 0.680–0.876) for identifying individuals with grade 2 and 3 hypertension. The index cut-off score for identifying individuals with grade 2 and 3 hypertension was 26/40, as indicated by the optimal match of Se and Sp (0.667 and 0.797, respectively) (Table 4, Figure 3).

## 4. Discussion

The ongoing increase in the prevalence of T2DM and HTN and their acknowledged impact on public health highlight the strain for more appropriate strategies and tools for their early diagnosis and prevention. Since both of these metabolic abnormalities may be provoked by the interaction of various risk factors relevant to family history, anthropometric indices and lifestyle parameters, such as dietary behaviors and physical activity, their assessment is necessary so as to design effective prevention strategies. Therefore, the development and implementation of screening tools evaluating holistically various health-related variables can be of a great importance in order to easily identify people at risk, so as to be referred for further evaluation at primary healthcare settings. The aim of the current study was to develop two risk assessment indices for the identification of IR and grade 2 and 3 HTN.

A number of risk scores to predict T2DM risk already exist in the literature [14,15,19,31], but most of them do not apply at various cases such as different races or socioeconomic status. One of the most reliable and valid tools for the identification of people at increased risk for T2DM is the FINDRISC [13], which was initially developed on the Finnish population, showing high Se and Sp values. Thereafter, it has been validated in other European ethnicities with good validity results [32,33,34,35]. Despite the fact that the FINDRISC has been designed on a specific population, it seems that the use of different cut-offs in different national groups may manage equivalent accuracy [36,37,38]. Similarly, an accurate easy-to-use online tool for the detection of impaired glucose regulation and T2DM is the Leicester Risk Assessment Score, an index developed in a multiethnic UK population with a high probability to identify population at risk [18]. Likewise, the Australian index AUSDRISK (The Australian Type 2 Diabetes Risk Assessment Tool) is considered of satisfactory Se and Sp but no studies have examined its validity in different ethnicities [14]. In general, the majority of tools available in the literature have been found to have satisfactory predictive values, but these values may be limited in the country developed [31] or some score components that are prerequisite for the completion of some tools such as blood indices measurements or the awareness of unfavorable glycaemia indices [13,15,19] makes them non-applicable when individuals have not previously undergone any blood testing.

The aforementioned tools are used for the assessment of the T2DM risk, the early identification of which is a major public health issue. Indeed, the early identification and management of IR may significantly decrease the risk for T2DM and cardiovascular disease (CVD) [39]. Furthermore, individuals in the highest HOMA-IR quintiles for IR have even greater risk for CVD [40]. The main benefit of pre-diabetes early identification is that it does not require medical management. The modification of lifestyle parameters, such as increase physical activity or decrease time devoted to sedentary activities, weight loss, adopting healthy dietary habits, etc., may be important measures in order to prevent the onset of glycaemic abnormalities [41,42,43,44], and thus to lower its economic public health burden [45].

Likewise, the existing risk scores for predicting HTN are either developed in population-specific groups or use as variables the measurements of systolic and diastolic BP [16], which has a large impact on the feasibility and thus the reach of these screening tools. For instance, the population cohort in the risk score of Kshirsagar et al. was middle-aged and older adults [17], and despite a satisfactory AUC (0.75–0.78), it is difficult to apply it in different age groups. Similarly, the risk index of the Strong Heart Study was developed in an American Indian population [23]. Despite the fact that obesity and hyperinsulinaemia affect less the BP of American Indians [46], it seems that the development of HTN in this ethnic group has a more severe effect on cardiovascular health [47]. One of the possible reasons for these findings in the American Indians is the presence of three genes with multiple single nucleotide polymorphisms associated with systolic BP [48]. Therefore, the development of more population-representative and self-reported risk scores is needed to identify HTN accurately.

In the current study, we developed two self-reported risk assessment indices, the European IR Risk Index and European HTN Risk Index for the identification of IR at 75th and 95th percentile according to HOMA-IR score and grade 2 and 3HTN. Both risk scores are calculated from a total of eleven components, and no biochemical, BP or other measurements are required. This makes these indices very easy-to-calculate, and applicable for a wide range of populations regardless their access to medical equipment, healthcare services, or their willingness to go through a medical check-up. Furthermore, by including anthropometric, dietary and physical activity components in the indices, the importance of lifestyle modification is highlighted. Specifically, the importance of increasing physical activity, along with breakfast and legume consumption, and of decreasing sugary drinks and alcohol consumption is highlighted. In addition, based on the ROC analyses, both the European IR Risk Index and the European HTN Risk Index were found to perform well in identifying individuals above 75th and 95th percentile of HOMA-IR and at grade 2 and 3 HTN (Table 4). The AUCs of 0.768, 0.828, 0.778, respectively, are slightly higher than the relevant of risk assessment indices available in the literature, which range from 0.72 to 0.78 [13,14,17,18,31,49], despite not incorporating biochemical and BP measurements. Another great advantage of the current indices is that the study population in which they have been developed and validated is from six countries in Europe, making them applicable and preferable for a wide range of Caucasian populations. The potential limitations of our study could be the fact that although it is based on a community cohort, the cohorts recruited from each country are not representative of the general population, as the recruitment took place only in one large region within each country, and with probably different risk of developing T2DM than the overall population according to FINDRISC. Overall, the findings are encouraging for the next steps in validating these risk assessment indices in more specific European populations.

## 5. Conclusions

T2DM and HTN are, without any doubt, the major risk factors for CVD. Moreover, there is robust evidence that they can be prevented and treated through lifestyle interventions. The disparity among populations around Europe has highlighted the need for simple novel screening tools, without the use of biochemical or other clinical indices in order to identify individuals at increased risk, so as to motivate them to seek medical assistance and proceed with lifestyle modification. The developed European IR Risk Index and European HTN Risk Index in the present study are easy-to-apply, valid, non-invasive, and low-cost for identifying European adults at high risk for developing T2DM or having HTN.

## Figures and Tables

**Table 1:** Descriptive characteristics of the European IR Risk Index and European HTN Risk Index.

	Development Cohort Mean ± SD	Validation Cohort Mean ± SD	*p* Value
European IR Risk Index (*n* = 1581)	*n* = 1076	*n* = 505	
Age (years)	40.7 ± 5.29	40.6 ± 5.15	0.666
BMI (kg/m^2^)			
male	29.7 ± 3.97	29.2 ± 4.39	0.172
female	27.3 ± 5.69	27.3 ± 5.67	0.985
Waist circumference (cm)			
male	102.7 ± 9.93	101.0 ± 11.64	0.068
female	88.7 ± 13.31	89.1 ± 12.95	0.619
HOMA-IR	2.0 ± 2.40	1.9 ± 1.39	0.340
SBP (mmHg)	116.8 ± 16.20	116.4 ± 15.47	0.673
DBP (mmHg)	77.7 ± 11.39	77.0 ± 10.37	0.242
European HTN Risk Index (*n* = 1350)	*n* = 906	*n* = 444	
Age (years)	40.1 ± 5.34	40.3 ± 5.47	0.590
BMI (kg/m^2^)			
male	29.2 ± 3.58	29.1 ± 3.89	0.224
female	27.1 ± 5.04	27.2 ± 5.48	0.930
Waist circumference (cm)			
male	102.8 ± 10.77	101.7 ± 12.07	0.330
female	87.6 ± 13.17	88.7 ± 13.41	0.268
HOMA-IR	2.2 ± 2.80	2.0 ± 1.46	0.145
SBP (mmHg)	117.5 ± 17.06	116.7 ± 16.51	0.466
DBP (mmHg)	77.9 ± 12.13	76.8 ± 11.06	0.092

BMI: Body Mass Index; HOMA-IR: Homeostatic Model Assessment of Insulin Resistance Index; SBP: Systolic blood pressure; DBP: Diastolic blood pressure.

**Table 2:** Scores for European IR Risk Index.

HOMA-IR Model	b	*p* Value	Cut-Offs	Points Allocated
BMI		0.001		
	-		<25 kg/m^2^	0
	0.340		25–30 kg/m^2^	9
	0.680		>30 kg/m^2^	19
Waist Circumference (women and men respectively)		0.003		
	-		<80 cm or <94 cm	0
	0.118		80–88 cm or 94–102 cm	3
	0.236		>88 cm or >102 cm	7
Screen time		0.001		
	-		<2 h/day	0
	0.113		≥2 h/day	3
Sex		0.023		
	-		female	0
	0.066		male	2
Breakfast		0.001		
	-		≥5 times/week	0
	0.095		<5 times/week	3
Sugary drinks (1 portion = 250 mL)		0.018		
	-		<1 portion/week	0
	0.063		≥1 portion/week	2
Walking (3 days/ week for at least 30 min)		0.033		
	-		Yes	0
	0.057		No	2
Vigorous physical activity (3 days/ week for at least 10 min)		0.002		
	-		Yes	0
	0.084		No	2
Maximum total points				40

b: standardizes-coefficient; BMI: Body Mass Index; HOMA-IR: Homeostatic Model Assessment of Insulin Resistance Index.

**Table 3:** Scores for European HTN Risk Index.

Hypertension Model	b	*p* Value	Cut-Offs	Points Allocated
BMI		0.001		
	-		<25 kg/m^2^	0
	0.308		25–30 kg/m^2^	10
	0.616		>30 kg/m^2^	20
Sex		0.001		
	-		female	0
	0.204		male	6
Vigorous physical activity (3 days/ week for at least 10 min)		0.091		
	-		Yes	0
	0.048		No	2
Legumes		0.001		
	-		≥1 cup/week	0
	0.254		<1 cup/week	8
Alcohol (1 portion = 125 mL of wine, 330 mL of beer or 40mL of hard liquor)		0.020		
	-		<3 portions/week	0
	0.069		≥3 portions/week	2
Age		0.099		
	-		<40 years	0
	0.047		≥40 years	2
Maximum total points				40

b: standardizes-coefficient; BMI: Body Mass Index; HTN: Hypertension.

**Table 4:** ROC characteristics of European IR Risk Index and European HTN Risk Index in the validation cohort.

	Score	AUC	95% Confidence Interval	*n* of TP	*n* of Un	PPV %	NPV %	Se	Sp
European IR Risk Index (*n* = 505)									
Cut off score for Identifying individuals above 75th percentile of HOMA-IR	23/40	0.768	0.721–0.815	95	37	45.5%	87.5%	0.720	0.691
Cut off score for Identifying individuals above 95th percentile of HOMA-IR	31/40	0.828	0.766–0.890	16	7	13.0%	98.2%	0.696	0.778
European HTN Risk Index (*n* = 444)									
Cut off for detecting 2nd and 3rd grade hypertension	26/40	0.778	0.680–0.876	14	7	14.0%	97.8%	0.667	0.797

AUC: Area under the ROC Curve, n of TP: Number of true positive, n of un: Number of unidentified, PPV: Positive predictive value, NPV: Negative predictive value, Se: Sensitivity, Sp: Specificity.
